# Mussel-inspired nanoparticle composite hydrogels for hemostasis and wound healing

**DOI:** 10.3389/fchem.2023.1154788

**Published:** 2023-03-30

**Authors:** Guihua Cui, Xiaoyu Guo, Ping Su, Tianshuo Zhang, Jiao Guan, Chungang Wang

**Affiliations:** ^1^ College of Chemistry, Northeast Normal University, Changchun, Jilin, China; ^2^ Department of Chemistry, Jilin Medical University, Jilin City, Jilin, China; ^3^ Jilin Vocational College of Industry and Technology, Jilin City, Jilin, China; ^4^ Affiliated 465 Hospital, Jilin Medical University, Jilin City, Jilin, China

**Keywords:** mussel-inspired, nanomaterials, hydrogels, hemostasis, wound healing

## Abstract

Uncontrolled hemorrhage caused by trauma can easily lead to death. Efficient and safe hemostatic materials are an urgent and increasing need for hemostatic research. Following a trauma, wound healing is induced by various cellular mechanisms and proteins. Hemostatic biomaterials that can not only halt bleeding quickly but also provide an environment to promote wound healing have been the focus of research in recent years. Mussel-inspired nanoparticle composite hydrogels have been propelling the development of hemostatic materials owing to their unique advantages in adhesion, hemostasis, and bacteriostasis. This review summarizes the hemostatic and antimicrobial fundamentals of polydopamine (PDA)-based nanomaterials and emphasizes current developments in hemorrhage-related PDA nanomaterials. Moreover, it briefly discusses safety concerns and clinical application problems with PDA hemostatic nanomaterials.

## 1 Introduction

Trauma is the leading cause of death among individuals aged 5–44 years (Krug et al., 2000; Behrens et al., 2014; Allison, 2019). Uncontrolled bleeding is a major cause of 30–40% of trauma deaths caused by war, traffic accidents, and natural disasters (Eastridge et al., 2011); in particular, the bleeding of irregular wounds, such as in the groin, is the weakest link in first aid. Massive prehospital blood loss also leads to higher mortality and serious complications later in life (nerve necrosis, amputation, etc.) (Kauvar et al., 2006). Therefore, new methods and products for effective bleeding control are the focus of research in the field of prehospital emergency care.

In recent years, many researchers have devoted themselves to the research and development of products applicable to wound hemostasis. There is a wide variety of existing hemostasis materials, particularly including polysaccharides, silicon-based materials, biological products, and self-assembled nano-peptides in various forms (including sponges, hydrogels, nano-fibers, and particles) (Chen et al., 2018a; Hickman et al., 2018; Lokhande et al., 2018; Wu et al., 2018; Du et al., 2020; Guo et al., 2021; Liang et al., 2021; Zhang et al., 2021; Wang et al., 2022a). The toxicity of degradation products, which induce immune responses and other safety problems, is a common problem with these materials. In addition, due to increased fluids and blood around the wound, the adhesion and biocompatibility of current hemostatic materials in moist environments must still be optimized. Therefore, the ideal hemostatic material is still the focus and difficulty of modern materials science research. In existing hemostatic materials, hydrogels have become the most competitive candidates for wound dressings due to their good hydrophilicity, biocompatibility, and three-dimensional (3D) porous structure that resembles extracellular matrix (ECM). Studies have shown that the mechanical properties of the hydrogels for wound healing are not only supported by physical sealing but also by the enrichment of coagulation factors through the absorption of wound extract (Liang et al., 2021).

In nature, mussels have excellent underwater adhesion. Byssus protein secreted by mussels has a fast curing speed, high waterproof adhesion ability, and excellent properties in water. Its adhesion diversity makes it a very advantageous and potential hemostatic material. There are six main mussel foot proteins (mfps) in mussel byssus, namely, mfp-1 through mfp-6. Phenolic residues including 3, 4-dihydroxyphenylalanine (DOPA), phenylalanine, and tyrosine have been found to be abundant in mussel byssus protein and play key roles in wet adhesion and adhesion diversification. Based on these discoveries, polydopamine (PDA) became the focus of attention as a novel coating material in 2007 due to its molecular structure, which is similar to DOPA (Lee et al., 2007). Since then, polydopamine has not been limited to use as a coating material but has also been rapidly incorporated into a wide range of applications across the biomedical field. Polydopamine has undergone intense interest in its applications and is becoming a material of global significance. There has thus been great interest in developing polydopamine-based hemostatic materials that target sites of wound bleeding to promote hemostasis. Both polydopamine and its derivative materials have been explored for the development of hemostatic and wound-healing materials. This review article provides an overview of polydopamine-based nanomaterial composite hydrogels used for hemostatic and antimicrobial fundamentals and emphasizes current developments in hemorrhage-related PDA nanomaterials. Moreover, it briefly discusses safety concerns and clinical application problems with PDA hemostatic nanomaterials.

## 2 Mechanism of hemostasis and wound healing promoted by mussel-inspired materials

**FIGURE 1:**
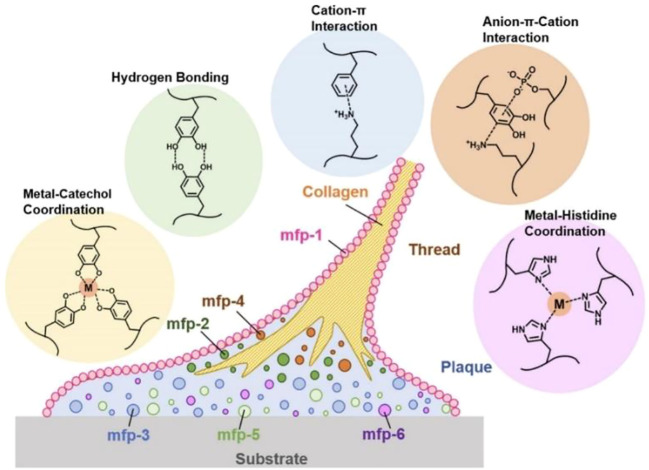
Phenolic residues of major mussel foot proteins (mfps) and typical, reversible mussel-inspired interactions (Chen and Zeng, 2022).

The tissue-adhesive properties of mussel-inspired materials are mainly attributable to mussel adhesion in the wet state. Mussels adhere to wet surfaces, and polyphenol compounds on mussel foot proteins play key roles (Maier et al., 2015; Guo et al., 2021). Polyphenol compounds contain large numbers of catechol groups, which act as adhesive reagents. Catechol functional groups contribute to tissue adhesion not only by participating in the formation of various reversible, non-covalent bonding forces (such as hydrogen bonding, π–π stacking, cation–π interaction, and coordination with metal oxides) (Ejima et al., 2013; Rodriguez et al., 2015; Gebbie et al., 2017; Waite, 2017; Patil et al., 2018), as shown in Figure 1 (Chen and Zeng, 2022), but also because polyphenol compounds are easily oxidized to quinone structures and have Schiff base bonds or Michael addition reactions with amino or sulfhydryl groups in histones. Catechol groups can also undergo coordination chelation reactions with metal ions such as Fe^3+^, form dynamic borate bonds with boric acid groups, and undergo disproportionation reactions to produce coupling to increase adhesion strength.

**FIGURE 2:**
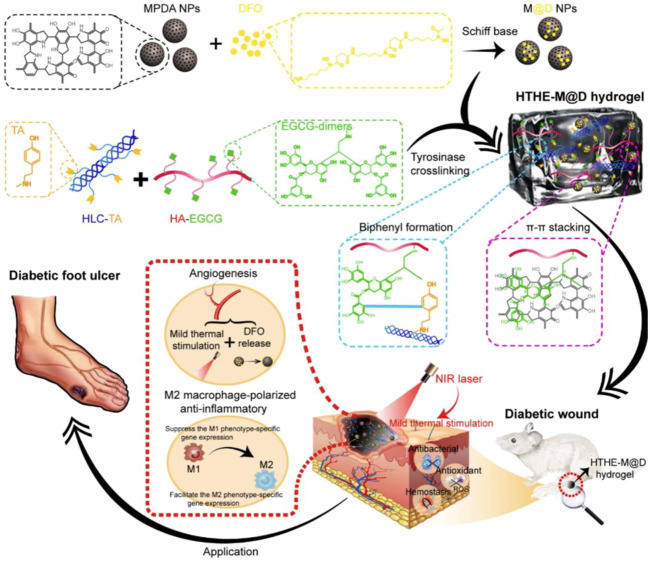
Schematic representation of the design strategy of the mussel-inspired hydrogel and mechanism of diabetic wound healing (Yuan et al., 2022).

Moreover, blood coagulation is accelerated by the interaction of catechol groups and the active residues of proteins or polysaccharides in the blood (Gopalakrishnan et al., 2014; Chen et al., 2018b; Geng et al., 2020a; Guo et al., 2021). In addition, the large amount of negative charge in the polyphenols can activate coagulation factor XII in the body, thus triggering its own coagulation cascade reaction and improving its hemostatic ability (Choi et al., 2014; Li et al., 2018a; Patil et al., 2018; Zhang et al., 2021). It was found that mussel polyphenol gel not only has strong adhesion but also forms a protective physical barrier through the high combination of polyphenol and protein, which prevents the invasion of foreign microorganisms and the normal growth of bacteria, thus playing a protective antibacterial function, as was confirmed by a preparation study of tannic acid–silk fibroin–diclofenac potassium (TA-SF-DP, TSD) hydrogels (Zhu et al., 2022). The TSD hydrogels effectively isolated ambient pollution, and the anti-inflammatory drug diclofenac potassium (DP) could be effectively released in the early traumatic stage to suppress local inflammation. Similar studies about the mechanisms of hemostasis and wound healing of polyphenol compounds in an M2 macrophage-polarized anti-inflammatory hydrogel (HTHE-M@D), combined with mild heat stimulation, were designed for DFU treatment, as shown in Figure 2 (Yuan et al., 2022). The HTHE-M@D hydrogel was prepared by the enzymatic cross-linking of epigallocatechin gallate dimer-grafted hyaluronic acid (HA-EGCG) and tyramine-grafted human-like collagen (HLC-TA), integrated with deferoxamine-loaded mesoporous polydopamine nanoparticles (M@D). The hydrogel exhibited a prominent enhancement of angiogenesis, which was attributed to the combination of mild heat stimulation *via* photothermal effects and angiogenic drugs (deferoxamine) released from the hydrogel. The hydrogel also promoted the transformation of macrophages from the M1 to the M2 phenotype and exhibited good anti-inflammatory, antibacterial, antioxidant and hemostatic properties, and biocompatibility. Additionally, the combination of hydrogel and mild thermal stimulation promoted skin regeneration in diabetic wounds and shortened the healing time to 13 days. This indicates that mussel-inspired materials have potential applications in the field of antibacterial hemostasis and wound healing.

## 3 Mussel-inspired nanoparticle composite hydrogels

Based on the mechanism of catechol groups, DOPA, dopamine (DA), 3,4-dihydroxyphenyl-propionic acid, and other compounds can form complex hemostatic materials with natural biomacromolecules including chitosan (Ryu et al., 2011; Shou et al., 2020), synthetic polymers such as polyethylene glycol (Bu et al., 2019; Zhao et al., 2020), and inorganic nanomaterials such as graphene oxide (Han et al., 2017a; Li et al., 2019). Mussel-inspired hemostatic hydrogels based on catechol groups can not only help stop bleeding but also keep the wound moist and contribute to wound healing (Wang et al., 2022b; Su et al., 2023). The amount, type, and activity of coagulation factors adsorbed on the surface of mussel-inspired hemostatic hydrogels determine the progress and efficiency of coagulation reactions. The unique properties of hemostatic nanomaterials are deeply attractive to researchers hoping to enhance availability and improve hemostatic effectiveness. Hemostatic nanomaterials have a larger surface area and produce nanoscale effects that promote blood absorption and cell adhesion; this is because hemostatic nanomaterials with large surface areas can quickly absorb a large amount of liquid such that the coagulation factor forms the reaction center surface on its surface, thus accelerating the blood coagulation process. Additionally, the adjustability of physical and chemical properties of hemostatic nanomaterials, such as size, surface, shape, and flexibility, make the hemostatic nanomaterials easier to customize for hemostatic devices in a variety of scenarios (Parani et al., 2016; Makabenta et al., 2021). The nanomaterials can promote wound healing, forming a kind of scaffold for cell growth. With the rapid development of nanotechnology and polymer materials science, inorganic or polydopamine nanoparticles could be dispersed into polymer networks through physical or chemical reactions to build mussel-inspired nanoparticle composite hydrogels with unique rheological properties of gel materials and nanomaterial functions, which could be adjusted to meet special mechanical requirements and have become an extremely important class of mussel-inspired hemostatic materials (Geng et al., 2020b; Guo et al., 2021; Wang et al., 2022a; Li et al., 2023). The PDA coating was used to assist in the synthesis of prominent antibacterial nanoparticles, silver nanoparticles (AgNPs), on the surface of the double-layer hydrogel (Li et al., 2022). The catechol group on PDA molecules can supply metal-binding sites and reduce Ag^+^ ions as a mild AgNP-reducing agent for unique chemical structures. The AgNP composite hydrogels exhibited high near-infrared (NIR) absorption at 808 nm, resulting in high temperature and NIR-enhanced peroxidase (POD)-like activity producing hydroxyl radicals (•OH), which endowed the hydrogels with excellent antibacterial properties when combined with released Ag^+^. At present, the mussel-inspired nanoparticle composite hydrogels that have been most studied include polyphenol–inorganic nanomaterial composite hydrogels and polydopamine nanoparticle composite hydrogels.

### 3.1 Polyphenol–inorganic nanomaterial composite hydrogels

Reversible, dynamic covalent and non-covalent linking interactions mediated by catechol groups have been optimized and used in the development of hemostatic materials. Graphene oxide (GO) and other nanomaterials are excellent combination for preparing polyphenol composite nanogels. To date, GO, carbon nanotubes (CNTs), nano-clay, hydroxyapatite, metal and metal oxide nanoparticles, and silicon-based nanomaterials have been reported to form nanocomposite hydrogels with dopamine and other analogs for hemostatic materials.

Nanomaterials such as GO nanosheets were reported to activate platelets and cause them to strongly aggregate to stop bleeding because the nanosheets were rich in oxygen-containing functional groups (Singh et al., 2011). GO can carry numerous other hemostatic factors when platelets are activated, adhere to wounds, and trigger clotting pathways. This property is similar to that of coagulants such as chitosan and thrombin (Howe and Cherpelis, 2013). However, previous studies on cross-linked graphene sponges have shown that these sponges lose their GO function to stimulate platelets due to the original GO being reduced under harsh reaction conditions (Li et al., 2018b). Compared with the cross-linked strategy, the mild self-polymerization of DOPA can retain the oxygen groups in the GO-based composite sponge. PDA cross-linked with GO using mild wet chemistry could retain oxygen-containing groups to further improve the hemostatic performance of GO sponges (Li et al., 2019). Furthermore, due to the random distribution and easy aggregation of nanomaterials in polymer networks, the hemostatic efficiency of nanomaterials can be reduced (Han et al., 2017a). Polyphenol compounds have an excellent binding ability with a variety of nanomaterials, which is conducive to the uniform distribution of nanomaterials in the hydrogel network, thus forming a new nanocomposite hydrogel (Wang et al., 2022b). Moreover, the successful deposition of compounds on the surface of nanomaterials could improve the antibleeding of the materials, such as for CNTs, the hydrophobicity limited their application in biomedical science (Shi et al., 2013; Wang et al., 2014). The polymer shell promoted the formation of uniform dispersion of CNTs in the aqueous medium, whereas ordinary carbon nanotubes were hindered by the strong van der Waals forces (Lynge et al., 2015).

Some studies indicate that using a polyphenol polymer layer as a coating could induce interfacial component synergy, change the interface properties of inorganic nanoparticles, and improve the adhesion of the materials to platelets and erythrocytes. For example, using PDA as a linker to immobilize thrombin on the surface of diatom biosilica diatom (DB-diatom) could maintain thrombin activity for a longer time (Mu et al., 2021), with thrombin activity being maintained at 67% for 30 days at room temperature. The composite materials quickly formed a fibrin network *in situ* and accelerated blood clot formation and strength to form a physical barrier at the wood. This was because the polyphenol structure forms highly active quinone structures that promote the covalent cross-linking between molecules and lead to irreversible and rapid curing. Similarly, PDA was inserted into the layers of two-dimensional layered nano-clay to form nanoaggregates and was crosslinked *in situ* with acrylamide monomer to form super-strong PDA–clay–PAM hydrogel (Han et al., 2017b). The PDA chains oxidized in the nano-space between the nano-clay layers maintained sufficient catechol groups to allow the adhesion of the hydrogel to persist for a long time. The hydrogel was reusable and durable, with adhesion remaining even under long-term storage conditions. Hydrogels could adhere to the surface of human skin and be non-irritating to the skin. Better yet, they peel off easily without causing any damage or pain to the skin during the peeling process.

Laponite (LAP) was reported as an effective hemostatic agent (Dawson and Oreffo, 2013). Incorporation of LAP nanoplates into the hydrogel could improve its properties and make it suitable for usage in hemorrhage. Mussel-inspired materials consisting of DOPA or PDA have been used to modify nano-clays in the composition of adhesive hydrogels to improve long-term mechanical stability as wound dressings (Liu et al., 2017; Rajabi et al., 2020; Ma et al., 2022a). The physical force between catechol and LAP could dissipate energy and improve the strength and toughness of nanomaterial composite hydrogels. Hydrogels have excellent malleability, and different shapes can be designed according to the needs of the wound. With the extension of time, the mussel-inspired materials part of the gel will form a covalent cross-linked structure to fix the shape of the tissue sealer, which is conducive to promoting healing of irregular wounds.

### 3.2 Polydopamine nanoparticle composite hydrogels

Synthetic melanin, often known as PDA, has good biocompatibility and can be biodegradable *in vivo*. Polydopamine nanoparticles (PDA-NPs) were reported to have excellent adhesion. PDA could be obtained by spontaneous oxidation or polymerization under alkaline conditions of DOPA, dopamine (DA), 5,6-dihydroxyindole (DHI), or other monomers (Liu et al., 2014). The size of PDA can be adjusted precisely by controlling experimental conditions such as reaction time, reaction temperature, reaction concentration, and reaction system pH value. Many PDA-based nanomaterials for hemostasis have been designed and synthesized in recent years (Liu et al., 2014; Qi et al., 2019; Tao et al., 2021; Ma et al., 2022b; Wang et al., 2022c; Sun et al., 2022), and PDA has been used as a bio-template, coating, or raw material to synthesize PDA-based nanomaterials.

**FIGURE 3:**
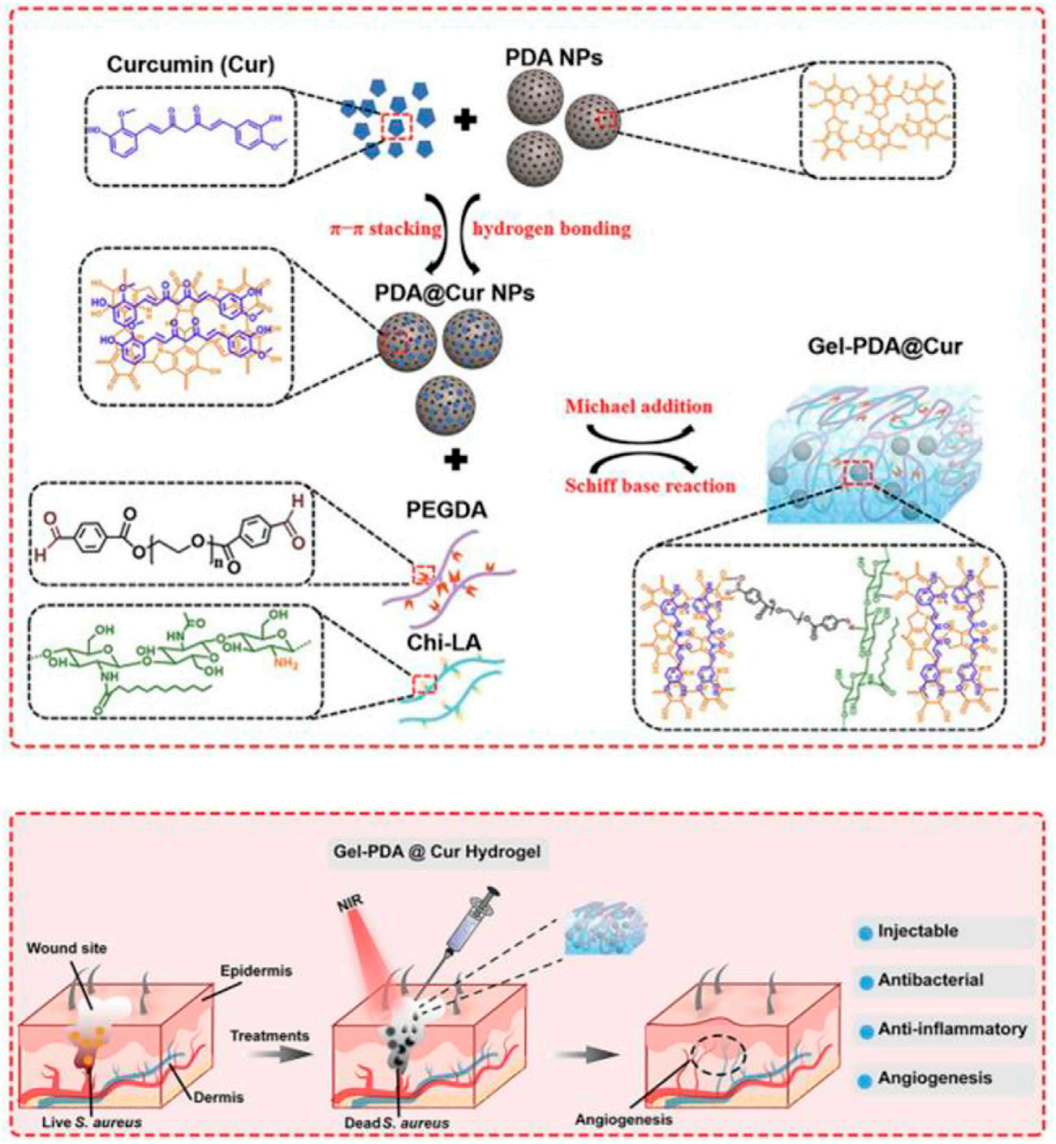
ynthesis route of Gel-PDA@Cur hydrogel and schematic representation of the fabricated hydrogel with NIR irradiation for bacterial inactivation to promote wound healing (Tao et al., 2021).

PDA with multiple catechol groups could be used as a convenient component for preparing anti-bleeding and self-healing materials. The unique properties of PDA-based nanomaterials were ascribed to the great balance between the reversible interactions (hydrogen bonding, π–π stacking, and hydrophobic interactions) and covalent bonds. Recently, a multifunctional near infrared (NIR) laser-induced hydrogel for infected wound healing was composed of dibenzaldehyde-grafted poly(ethylene glycol) (PEGDA), lauric acid-terminated chitosan (Chi-LA), and curcumin (Cur)-loaded mesoporous polydopamine nanoparticles (PDA@Cur) *via* Schiff base and/or Michael addition reaction, as shown in Figure 3 (Tao et al., 2021). NIR irradiation could activate the photothermal PDA NPs in Gel-PDA@Cur hydrogel and generate local hyperthermia for antibiosis. *In vivo* treatment of *staphylococcus aureus* infection in a full-layer skin defect model showed that the Gel-PDA@Cur hydrogel had a good hemostatic function and excellent wound healing ability.

Other nanoparticles that, like PDA, have a controlled size were combined with mussel-inspired hyaluronic acid (HA) hydrogels to form a nanocomposite (Qi et al., 2019). Compared with other nanoparticles such as f poly (lactic-co-glycolic acid) (PLGA) and PLGA-(*N*-hydroxysuccinimide) (PLGA-NHS) nanoparticles, the optimal lap shear strength (47 ± 3 kPa d) was achieved by combining PDA nanoparticles of 200 nm (12.5% w/v) with an HA hydrogel (40% w/v); increased adhesion strength was induced by including PDA nanoparticles in nanocomposites. Here, the multi-functional mussel-inspired nanoparticles composites hydrogels were fabricated *via in-situ* polymerization by using PDA-NPs and *N*-isopropylacrylamide (NIPAM) (Nikhil et al., 2022). PDA-NPs were attached to the hydrogel surface as a coating. The strong tissue adhesion of hydrogels was due to a large number of catechol groups. The experimental results showed that the hydrogels had a synergistic ability to promote wound healing. It has been reported that the stability of hydrogels can be enhanced at low temperatures by decreasing their temperature sensitivity.

## 4 Conclusion and outlook

Compared with natural hemostatic components, mussel-inspired nanoparticle composite hydrogels can more easily form tissue adhesion and promote wound healing. Results from a bacteria-infected skin defect remolding experiment showed that the PDA @AgNP-based composite hydrogels thicken granulation tissue due to the hydrogel providing a satisfying wound environment (Geng et al., 2020b). Something like that the gelatin was firstly functionalized by dopamine to form dopamine-grafted gelatin (GelDA) and mixed with 1,4-phenylenebisboronic acid and graphene oxide (GO) to obtain GelDA/GO hydrogels (Han et al., 2016). The adhesion strength of hydrogels was higher than that of commercial dressings (about 5 kPa) and was strong enough to close the wound and stop the bleeding. The reversible molecular interaction of polyphenols gives mussel-inspired materials universal adhesion and self-healing behavior. With comprehensive studies on compounds with polyphenols, reversible interactions (including hydrogen bonding, metal–catechol coordination, metal–histidine coordination, hydrophobic interactions, and cation–π, anion–π, and π–π interactions) mediated by catechol or other groups have been discovered and elucidated (Han et al., 2021). PDA and other natural polyphenols with multiple phenolic groups have great research value and application prospects as building blocks for hemostatic and self-healing materials.

The adhesion of polyphenol hydrogels is significantly affected by the content of free phenol hydroxyl groups in the hydrogels. The phenol hydroxyl group moieties would be oxidized by high temperatures or free radicals, tremendously reducing the adhesion of the hydrogels (Liang et al., 2021). In practical application, the long-term stability of adhesives and the change of color and adhesion strength caused by oxidation should be considered. It is very important to establish a simple and effective method of incorporating PDA or polyphenols into other materials and retaining their properties for the preparation of novel polyphenol hemostatic agents. On the other hand, the hemostasis of polyphenol composite hydrogels is a synergistic process, and how to find appropriate methods and determine the interactions of different groups is extremely important for understanding the synergistic effects of different interactions in mussel-inspired nanoparticle composite hydrogels (Yan et al., 2021). In addition, the size of polydopamine nanoparticles is critical in the design of delivery systems with specific targets in wound healing. In recent years, studies on the size, structure, and biological applications of PDA nanoparticles, especially hemostasis, have been ongoing (Lemaster et al., 2019; Salomaki et al., 2019; Vasileiadis et al., 2022). Meanwhile, the adhesive ability of hemostatic hydrogels is also a disadvantage for wound healing. The principal problem is how to remove hydrogels that adhere strongly to wounds without causing secondary pain and bleeding (Guo et al., 2021; Liang et al., 2021). The swelling rate of the hydrogel affects the ability of the wound dressing to absorb the wound exudate, thereby reducing the risk of bacterial infection forming (Qi et al., 2019). The swelling ratio of PDA @AgNP-based hydrogels was 228% ± 9.44%, indicating that these hydrogels exhibit a suitable water-absorbing ability. With the development of science and technology, there have been numerous indicators for evaluating the efficiency of hydrogels, including tensile mechanical properties and rheological behavior, shear-thinning performance and adhesiveness, photothermal properties and antibacterial activity, hemocompatibility, and *in vivo* hemostatic performance and cytocompatibility. Mussel-inspired nanoparticle composite hydrogels, as hemostatic materials integrating adhesion, antibleeding, and bacteriostasis, will be improved and developed more in the field of hemostasis and will realize clinical applications.
